# Association of Lactate‐to‐Albumin Ratio With 28‐Day Mortality in Critically Ill Patients With Obstructive Sleep Apnea: A Retrospective Cohort Study Based on the MIMIC‐IV Database

**DOI:** 10.1155/carj/6582764

**Published:** 2026-11-05

**Authors:** Jian Zhou, Chuntian Chi, Fanghao Yu, Junpei Wu, Hanjian Lou

**Affiliations:** ^1^ Department of Respiratory and Critical Care Medicine Yiwu Central Hospital Yiwu Zhejiang China ywzxyy.com; ^2^ Department of Laboratory Medicine Yiwu Central Hospital Yiwu Zhejiang China ywzxyy.com

**Keywords:** 28-day mortality, biomarker, critically ill, lactate-to-albumin ratio, MIMIC-IV database, OSA, risk factor

## Abstract

**Background:**

Obstructive sleep apnea (OSA) is not uncommon among critically ill patients, especially in those with acute hypercapnic respiratory failure. Its high mortality rate remains a major challenge in the intensive care setting. The frequent apnea and respiratory failure during sleep in OSA patients, along with the intermittent hypoxemia caused by it, are accompanied by a series of systemic pathological and physiological changes. Metabolic imbalance is a key characteristic of the underlying pathological mechanism of this disease. However, the association between lactate‐to‐albumin ratio (LAR) and the prognosis of OSA patients remains to be fully elucidated. To date, a paucity of studies has evaluated the prognostic capabilities of these indicators. The majority of these studies have focused on the neutrophil‐to‐lymphocyte ratio (NLR) and platelet‐to‐lymphocyte ratio (PLR), and the results are contradictory, insufficient to fully clarify their clinical significance. Relying solely on lactate may not fully reflect patients’ physiological conditions, whereas integrating LAR can provide a more comprehensive and accurate description of the metabolic state. This study aims to explore the relationship between LAR and 28‐day mortality rates in ICU patients with OSA, using data from the MIMIC‐IV database.

**Methods:**

Patients diagnosed with OSA were selected from the Medical Information Mart for Intensive Care IV (v.2.2) database, identified by specific ICD‐9 and ICD‐10 codes (detailed in the Methods section). To assess the predictive power of LAR, receiver operating characteristic (ROC) curve analysis, Kaplan–Meier (K‐M) survival curve analysis, logistic regression analysis, and subgroup analysis were employed. LAR was analyzed both as a categorical variable (based on the optimal cutoff) and as a continuous variable.

**Results:**

The sample population comprised 807 patients diagnosed with OSA who were admitted to the intensive care unit for the first time. The 28‐day mortality rate was 20.44%. The area under the ROC curve (AUC) for LAR was 0.730 (95% CI: 0.686–0.772), which was comparable with OASIS (0.736; 95% CI: 0.695–0.776) and superior to SOFA (0.637; 95% CI: 0.591–0.683; DeLong test *p* = 0.500). The optimal cutoff value for LAR was 0.791. The high‐LAR group demonstrated a poor prognosis in the K‐M analysis (*p* < 0.001). Multivariate logistic regression demonstrated a significant association between LAR and unfavorable outcomes, with an odds ratio (OR) of 3.00 (95% CI: 1.83–4.90, *p* < 0.001) when analyzed as a dichotomous variable, and an OR of 1.52 per unit increase (95% CI: 1.27–1.82, *p* < 0.001) when analyzed as a continuous variable. Subgroup analysis revealed no statistically significant interaction between LAR and any subgroup (*p* for interaction: 0.052–0.906).

**Conclusion:**

LAR is a rational and easily accessible biomarker that is significantly associated with 28‐day mortality in ICU patients with OSA. These findings are only applicable to OSA patients who received lactate and albumin testing during ICU admission, and the results cannot be generalized to the entire population of ICU patients with OSA.

## 1. Introduction

Obstructive sleep apnea (OSA) is a common disease, estimated to affect nearly one billion people worldwide, with its incidence increasing with age [1]. OSA is characterized by partial or complete obstruction of the upper airway, intermittent hypoxia, and frequent arousals from sleep in recurrent episodes [2]. The intermittent hypoxemia caused by frequent apnea and hypopnea during sleep in patients with OSA is accompanied by a series of systemic pathological and physiological changes [3]. It often leads to excessive daytime sleepiness (EDS), which may adversely affect various aspects of daily life, including performance, emotional state, and overall well‐being [4–6]. These pauses in breathing can lead to reductions in arterial blood oxygen saturation (SaO_2_) and tissue hypoxia. After the breathing pause ceases, reoxygenation occurs. This cycle drives the evolution of hypoxic oxidative stress (HOS). HOS has been demonstrated to trigger excessive glucose degradation through the glycolytic pathway, resulting in pyruvate accumulation [7]. Lactate dehydrogenase is the enzyme responsible for converting accumulated pyruvate into lactate. In the hypoxemic state, the lack of ATP significantly impairs hepatic lactate utilization (Cori cycle) and renal cortical gluconeogenesis. Therefore, tissue hypoxia leads to hyperlactatemia and lactic acidosis [8, 9].

A preponderance of earlier research has determined that lactate exhibits the most robust correlation with the mortality rate of critically ill patients [10, 11]. OSA is prevalent among critically ill patients and associated with increased mortality [12]. Moreover, the prevalence of malnutrition among OSA patients is often attributed to the presence of chronic diseases. A diagnosis of malnutrition is made on the basis of an albumin level of less than 3.5 g/dL. In patients receiving intensive care, low serum albumin levels (less than 3 g/dL) have been demonstrated to be associated with elevated in‐hospital mortality rates in comparison with higher levels, with an odds ratio (OR) of 1.51 [13]. Therefore, lactic acid and albumin may be closely related to the outcomes of patients with OSA. Recent studies have found that the lactate‐to‐albumin ratio (LAR) is associated with the clinical outcomes of critically ill patients [14], spontaneous subarachnoid hemorrhage [15], and community‐acquired pneumonia [16]. However, the extent to which LAR holds prognostic significance in patients diagnosed with OSA who are admitted to intensive care units (ICUs) remains unclear.

Therefore, in this study, we explored the relationship between LAR and 28‐day mortality rates in ICU patients with OSA, utilizing data from the MIMIC‐IV database. To evaluate its potential clinical utility in distinct patient subsets, we conducted a comprehensive analysis of its predictive power within these defined groups.

## 2. Methods

### 2.1. Database

The study cohort was drawn from the MIMIC‐IV database (Version 2.2), which includes anonymous medical records of patients admitted to ICUs at Beth Israel Deaconess Medical Center (2008–2019). The database contains records relating to a variety of factors, including but not limited to demographics, vital signs, clinical laboratory tests, fluid balance data, and information regarding the patient’s overall status. OSA cases were identified using the following ICD codes: ICD‐9‐CM codes 327.23 (OSA) and 780.57 (sleep apnea, unspecified); and ICD‐10‐CM codes G47.33 (OSA [adult]), G47.30 (Sleep apnea, unspecified), and G47.39 (Other sleep apnea). The critical care team’s manual verification of data from bedside monitoring systems provided reliable, verifiable evidence of patients’ physiological states. One author (Chuntian Chi) successfully completed the CITI Data or Specimens Only Research course and obtained database access certification (certification number 66935858). The research project was conducted in strict accordance with the ethical guidelines stipulated in the Declaration of Helsinki (2013 revision). Ethical approval for the use of the MIMIC database was granted by the Institutional Review Boards of both the Massachusetts Institute of Technology and Beth Israel Deaconess Medical Center. Both agreements eliminate the necessity for participants to provide explicit consent when engaging in research utilizing the MIMIC‐IV dataset; thus, our study did not require additional ethics committee approval from our own center.

### 2.2. Study Population

Participants were drawn from the MIMIC‐IV database. From 2008 to 2019, a total of 431,231 patients were registered in the database. We first excluded patients admitted to the ICU more than once (*n* = 380,311), leaving those with a first ICU admission. A total of 30,371 patients diagnosed with OSA who received their first ICU treatment between 2008 and 2019 were identified. Subsequently, 29,564 patients with missing lactate or albumin values were excluded, resulting in a final cohort of 807 patients. The missing data pattern was primarily attributable to the fact that lactate and albumin are not measured as part of routine admission panels for every ICU patient; instead, they are ordered at the discretion of the treating clinician, typically for patients with suspected tissue hypoperfusion, metabolic disturbance, or nutritional concerns. The excluded group had a lower overall severity of illness on average, suggesting that our cohort may overrepresent sicker patients; this potential selection bias is acknowledged in the Limitations section. The screening process is summarized in Figure 1.

**FIGURE 1:**
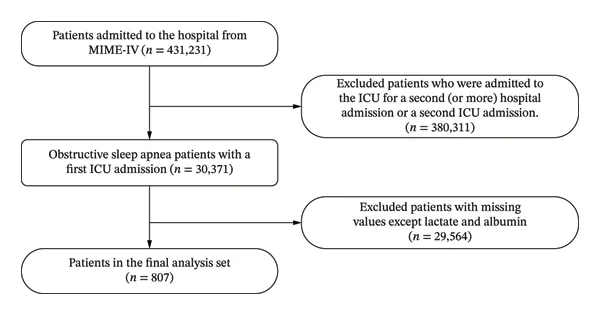
The flowchart of the screening process. Abbreviations: ICU: intensive care unit, MIMIC‐IV: Medical Information Mart for Intensive Care‐IV study cohort. Illustration of the exclusion and inclusion criteria utilized to select the final cohort of 807 patients.

### 2.3. Data Extraction

To facilitate data extraction and management, we employed Structured Query Language (SQL), specifically PostgreSQL Version 9.6, alongside STATA Version 17.0. Information encompassing patient age, gender, pre‐existing health conditions, initial lab results upon ICU admission, first‐day severity of illness (Sequential Organ Failure Assessment [SOFA], Oxford Acute Severity of Illness [OASIS], Simplified Acute Physiology Score II [SAPSII]), mechanical ventilation (MV) status on ICU arrival, duration of hospitalization and ICU stay, and dates of death were extracted. Clinical comorbidities identified by ICD codes included myocardial infarction, congestive heart failure, peripheral vascular disease, diabetes, renal disease, malignant cancer, AIDS, cerebrovascular disease, chronic pulmonary disease, and severe liver disease. On the first day, the following laboratory tests were recorded: arterial blood gases (pH, PaCO_2_, PaO_2_, and base excess), white blood cells, platelets, total bilirubin, hematocrit, hemoglobin, creatinine, glucose, calcium, chloride, potassium, sodium, lactate, and albumin. First‐day vital signs included heart rate, respiratory rate, mean arterial blood pressure, and SpO_2_. Where patients had multiple measurements for a given variable on the first ICU day, the earliest value was utilized.

### 2.4. Statistical Analysis

Normally distributed continuous variables are reported as mean ± standard deviation (SD); nonnormally distributed variables are reported as median and interquartile range (IQR). Categorical variables are expressed as frequencies and percentages (%). Differences in continuous variables between groups were assessed using the independent‐samples *t*‐test or Mann–Whitney *U* test, as appropriate; categorical variables were compared using the chi‐squared test or Fisher’s exact test.

The primary outcome was 28‐day mortality following ICU admission. To assess the association between LAR and mortality, we employed both univariate and multivariate logistic regression models, reporting OR with 95% confidence intervals (CIs). LAR was analyzed both as a continuous variable (per‐unit increase) and as a dichotomous variable based on the optimal cutoff derived from the receiver operating characteristic (ROC) curve. For variable selection in the multivariate model, we adopted a two‐step strategy: First, all variables with *p* < 0.05 in univariate analysis were entered into the initial model; second, clinically important variables (age and gender) were retained regardless of univariate significance to address potential confounding and improve clinical interpretability. Clinically meaningful indicators including SOFA and OASIS scores were retained on the basis of clinical relevance rather than merely statistical significance. Collinearity diagnostics were performed prior to multivariate regression modeling. Given that LAR was derived from lactate and albumin, raw lactate and albumin values were intentionally excluded from models containing LAR to avoid multicollinearity. This approach balances data‐driven selection with clinical judgment.

ROC curves were constructed to evaluate the discriminatory performance of LAR, SOFA, and OASIS for predicting 28‐day mortality. The area under the ROC curve (AUC) with 95% CI was calculated for each indicator. Pairwise comparisons of AUCs were performed using the DeLong test, and the resulting *p* values indicate whether the discriminatory abilities of the two compared indicators differ statistically. The optimal cutoff value for LAR was determined using Youden’s index. Based on this threshold, patients were categorized into high‐LAR and low‐LAR groups.

Kaplan–Meier survival curves were generated and compared using the log‐rank test. Subgroup analyses with interaction tests were conducted to explore whether the association between LAR and 28‐day mortality varied across comorbidity strata.

All statistical tests were two‐sided, and a *p* value < 0.05 was considered statistically significant. For *p* value reporting, we followed standard conventions: *p* < 0.001 is reported as “*p* < 0.001,” *p* values between 0.001 and 0.01 are reported to three decimal places, *p* values ≥ 0.01 and < 0.99 are reported to two decimal places, and *p* > 0.99 is reported as “*p* > 0.99.” All analyses were performed using R Version 4.2.1 (R Foundation for Statistical Computing, Vienna, Austria) with the following packages: pROC for ROC analysis, survival for Kaplan–Meier and regression models, and rms for regression modeling. The study was conducted in accordance with the STrengthening the Reporting of OBservational studies in Epidemiology (STROBE) guidelines.

## 3. Results

A total of 807 patients were included in this study. The median age of the study population was 64.8 years. Male subjects accounted for 66% of the total study population. The 28‐day mortality rate was 20.44% (165 of 807 patients). Compared with survivors, nonsurvivors were older and had higher SOFA and OASIS scores. Additionally, white blood cell count, BUN, creatinine, glucose, and LAR were higher in the deceased group. Detailed baseline characteristics are presented in Table 1.

**TABLE 1:** Baseline characteristics between survivors and nonsurvivors.

Independent variables	Total (*n* = 807)	Survivors (*n* = 642)	Death (*n* = 165)	*p* value
Demographics				
Gender				0.857
M	533 (66.0)	425 (66.2)	108 (65.5)	
F	274 (34.0)	217 (33.8)	57 (34.5)	
Age (years)	64.8 ± 12.7	63.9 ± 12.6	68.5 ± 12.3	< 0.001
Comorbidities				
Myocardial infarct				0.043
0	660 (81.8)	534 (83.2)	126 (76.4)	
1	147 (18.2)	108 (16.8)	39 (23.6)	
Congestive heart failure				0.153
0	402 (49.8)	328 (51.1)	74 (44.8)	
1	405 (50.2)	314 (48.9)	91 (55.2)	
Peripheral vascular disease				0.343
0	698 (86.5)	559 (87.1)	139 (84.2)	
1	109 (13.5)	83 (12.9)	26 (15.8)	
Dementia				0.044
0	787 (97.5)	630 (98.1)	157 (95.2)	
1	20 (2.5)	12 (1.9)	8 (4.8)	
Cerebrovascular disease				0.131
0	712 (88.2)	572 (89.1)	140 (84.8)	
1	95 (11.8)	70 (10.9)	25 (15.2)	
Chronic pulmonary disease				0.33
0	472 (58.5)	370 (57.6)	102 (61.8)	
1	335 (41.5)	272 (42.4)	63 (38.2)	
Rheumatic disease				0.05
0	771 (95.5)	618 (96.3)	153 (92.7)	
1	36 (4.5)	24 (3.7)	12 (7.3)	
Peptic ulcer disease				0.576
0	782 (96.9)	621 (96.7)	161 (97.6)	
1	25 (3.1)	21 (3.3)	4 (2.4)	
Mild liver disease				0.164
0	670 (83.0)	539 (84)	131 (79.4)	
1	137 (17.0)	103 (16)	34 (20.6)	
Diabetes				0.099
0	498 (61.7)	387 (60.3)	111 (67.3)	
1	309 (38.3)	255 (39.7)	54 (32.7)	
Paraplegia				0.62
0	772 (95.7)	613 (95.5)	159 (96.4)	
1	35 (4.3)	29 (4.5)	6 (3.6)	
Renal disease				0.007
0	518 (64.2)	427 (66.5)	91 (55.2)	
1	289 (35.8)	215 (33.5)	74 (44.8)	
Malignant cancer				0.094
0	706 (87.5)	568 (88.5)	138 (83.6)	
1	101 (12.5)	74 (11.5)	27 (16.4)	
Severe liver disease				0.001
0	747 (92.6)	604 (94.1)	143 (86.7)	
1	60 (7.4)	38 (5.9)	22 (13.3)	
Aids				1
0	804 (99.6)	639 (99.5)	165 (100)	
1	3 (0.4)	3 (0.5)	0 (0)	
Severity of illness				
SOFA	4.0 (2.0, 5.0)	3.0 (2.0, 5.0)	4.0 (3.0, 7.0)	< 0.001
OASIS	37.0 (31.0, 44.0)	36.0 (30.0, 42.0)	44.0 (38.0, 50.0)	< 0.001
SAPSII	42.0 (34.0, 51.5)	40.0 (32.0, 48.0)	55.0 (44.0, 65.0)	< 0.001
Vital signs				
HR (bpm)	106.0 (91.0, 120.0)	104.0 (90.8, 119.0)	113.0 (97.0, 131.0)	< 0.001
RR (bpm)	29.0 (25.0, 33.0)	29.0 (25.0, 33.0)	30.0 (27.0, 35.0)	0.003
MBP (mmHg)	75.3 (70.0, 81.5)	75.8 (70.7, 82.6)	73.3 (66.8, 78.0)	< 0.001
SpO_2_ (%)	100.0 (99.0, 100.0)	100.0 (99.0, 100.0)	100.0 (99.0, 100.0)	0.939
Laboratory results				
WBC (109/L)	14.4 (10.1, 20.0)	14.1 (10.1, 19.2)	16.2 (10.1, 22.4)	0.024
Platelet (109/L)	213.0 (157.0, 292.5)	218.0 (161.2, 297.8)	195.0 (147.0, 273.0)	0.006
Hematocrit (%)	35.4 (30.4, 40.5)	35.7 (30.6, 40.4)	34.0 (29.1, 40.8)	0.261
Hemoglobin (g/dL)	11.4 (9.6, 13.0)	11.4 (9.7, 13.1)	11.1 (9.2, 12.8)	0.116
Bun (mg/dL)	31.0 (20.0, 51.0)	28.0 (19.0, 46.8)	45.0 (29.0, 64.0)	< 0.001
Glucose (mg/d)	172.0 (134.0, 236.0)	169.0 (133.0, 228.0)	185.0 (143.0, 263.0)	0.048
Creatinine (mg/dL)	1.6 (1.0, 2.7)	1.4 (1.0, 2.5)	2.3 (1.4, 3.4)	< 0.001
Sodium (mmol/L)	140.0 (137.0, 143.0)	140.0 (138.0, 143.0)	140.0 (137.0, 143.0)	0.362
Calcium (mmol/L)	8.6 (8.1, 9.1)	8.6 (8.1, 9.1)	8.6 (8.2, 9.2)	0.321
Chloride (mmol/L)	105.0 (100.0, 108.0)	105.0 (100.0, 108.0)	104.0 (98.0, 109.0)	0.385
Potassium (mmol/L)	4.6 (4.2, 5.4)	4.6 (4.1, 5.3)	4.9 (4.3, 5.7)	0.003
PO_2_	137.0 (88.5, 237.0)	134.5 (88.0, 228.8)	144.0 (94.0, 262.0)	0.232
PCO_2_	48.0 (42.0, 60.0)	47.5 (42.0, 60.0)	49.0 (41.0, 61.0)	0.693
PH	7.4 (7.3, 7.4)	7.4 (7.3, 7.4)	7.4 (7.3, 7.4)	0.009
Base excess	0.0 (−2.0, 4.0)	0.0 (−2.0, 4.0)	0.0 (−5.0, 2.0)	< 0.001
LAR	0.6 (0.4, 1.1)	0.5 (0.4, 0.9)	1.1 (0.6, 2.5)	< 0.001

*Note:* OASIS, Oxford Acute Disease Severity Score; MBP, mean arterial blood pressure.

Abbreviations: HR, heart rate; LAR, lactate‐to‐albumin ratio; RR, respiratory rate; SAPSII, Simplified Acute Physiology Score II; SOFA, Sequential Organ Failure Assessment; WBC, white blood cells.

**TABLE 2:** Baseline characteristics of included and excluded OSA patients.

Independent variables	Included cohort (*n* = 807)	Excluded cohort (*n* = 29,564)	*p* value
Demographics			
Age, years, median (IQR)	64.8 (52.1,76.3)	61.2 (49.5,73.0)	< 0.001
Male, *n* (%)	533 (66.0%)	18,812 (63.6%)	0.072
Comorbidities, *n* (%)			
Myocardial infarction	147 (18.2)	4918 (16.6)	0.143
Congestive heart failure	402 (49.8)	14,025 (47.4)	0.13
Peripheral vascular disease	109 (13.5)	3708 (12.5)	0.343
Dementia	20 (2.5)	946 (3.2)	0.187
Cerebrovascular disease	95 (11.8)	3252 (11.0)	0.401
Chronic pulmonary disease	335 (41.5)	12,043 (40.7)	0.526
Rheumatic disease	36 (4.5)	1183 (4.0)	0.462
Peptic ulcer disease	25 (3.1)	857 (2.9)	0.769
Mild liver disease	137 (17.0)	4730 (16.0)	0.366
Diabetes	309 (38.3)	11,214 (37.9)	0.784
Paraplegia	35 (4.3)	1301 (4.4)	0.905
Renal disease	289 (35.8)	9056 (30.6)	< 0.001
Malignant cancer	101 (12.5)	3420 (11.6)	0.337
Severe liver disease	60 (7.4)	1655 (5.6)	0.017
AIDS	3 (0.4)	104 (0.4)	0.977
Illness severity scores, median (IQR)			
SOFA	4.0 (2.0,5.0)	2.0 (1.0,3.0)	< 0.001
OASIS	37.0 (31.0,44.0)	30.0 (25.0,36.0)	< 0.001
SAPSII	42.0 (34.0,51.5)	33.0 (26.0,41.0)	< 0.001
Vital signs, median (IQR)			
HR (bpm)	106 (91,120)	94 (82,108)	< 0.001
RR (bpm)	29 (25,33)	24 (20,29)	< 0.001
MBP (mmHg)	75.3 (70.0,81.5)	82.1 (76.0,88.5)	< 0.001
SpO2 (%)	100 (99,100)	100 (99,100)	0.614
Laboratory indicators, median (IQR)			
WBC (10^9^/L)	14.4 (10.1,20.0)	10.2 (7.1,14.6)	< 0.001
Platelet (10^9^/L)	213 (157,292)	226 (168,305)	0.003
Creatinine (mg/dL)	1.6 (1.0,2.7)	1.1 (0.8,1.8)	< 0.001
BUN (mg/dL)	31 (20,51)	22 (14,33)	< 0.001
Glucose (mg/dL)	172 (134,236)	145 (112,194)	< 0.001
Lactate (mmol/L)	1.3 (0.9,2.2)	— (missing)	—
Albumin (g/dL)	3.1 (2.6,3.6)	— (missing)	—
LAR	0.6 (0.4,1.1)	— (missing)	—

*Note:* Continuous variables: median (interquartile range), analyzed using the Mann–Whitney *U* test; categorical variables: *n* (%), analyzed using chi‐square test. *p* < 0.05 indicates statistical significance. Table 2 confirms that excluded patients had milder conditions, suggesting that the selective testing of lactate/albumin in only severe cases introduced selection bias. OASIS, Oxford Acute Disease Severity Score; MBP, mean arterial blood pressure.

Abbreviations: HR, heart rate; LAR, lactate‐to‐albumin ratio; RR, respiratory rate; SAPSII, Simplified Acute Physiology Score II; SOFA, Sequential Organ Failure Assessment; WBC, white blood cells.

### 3.1. ROC Curve Analysis and Kaplan–Meier Curve

We plotted the ROC curves of three indicators—LAR, SOFA, and OASIS—for predicting 28‐day mortality in ICU patients with OSA, as shown in Figure 2. The AUC of LAR (0.730; 95% CI: 0.686–0.772) was comparable with OASIS (0.736; 95% CI: 0.695–0.776; DeLong test *p* = 0.361) and superior to SOFA (0.637; 95% CI: 0.591–0.683; DeLong test *p* = 0.270). Detailed results are listed in Table 3.

**FIGURE 2:**
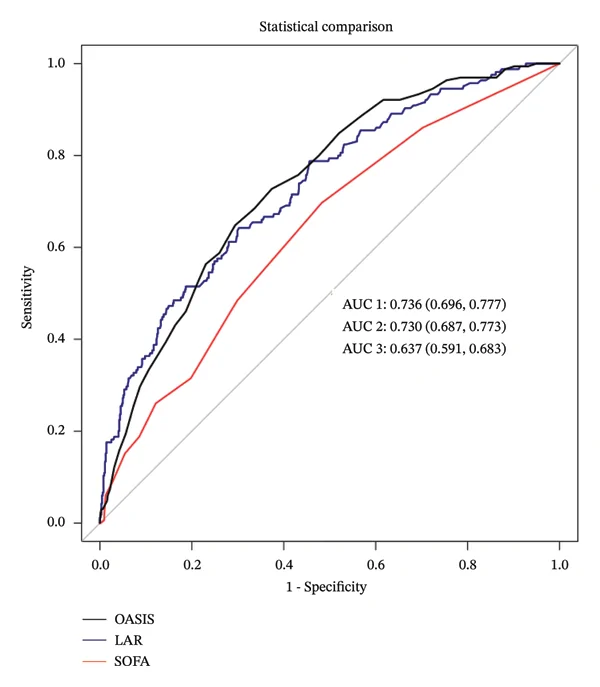
A comparison of the ROC curves for predicting 28‐day mortality using LAR, SOFA, and OASIS scores.

**TABLE 3:** ROC curve findings for predicting 28‐day mortality.

Variable	AUC	95% CI	Cut‐off	Sensitivity	Specificity	*p* value (DeLong vs. LAR)
LAR	0.730	0.686–0.772	0.791	0.642	0.697	—
SOFA	0.637	0.591–0.683	3.5	0.697	0.517	0.270
OASIS	0.736	0.695–0.776	40.5	0.648	0.705	0.361

*Note:* The optimal LAR cutoff value determined by Youden’s index was 0.791. Accordingly, patients were categorized into the low‐LAR group (LAR < 0.791, *n* = 507) and the high‐LAR group (LAR ≥ 0.791, *n* = 300). As shown in Figure 3, the Kaplan–Meier survival curve demonstrated that the prognosis in the high‐LAR group was significantly poorer than that in the low‐LAR group (log‐rank *p* < 0.001).

**FIGURE 3:**
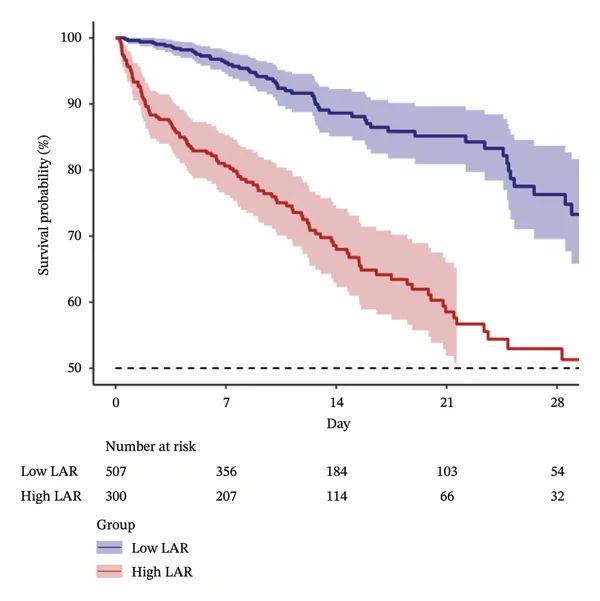
The following study will examine the Kaplan–Meier survival curves for patients with obstructive sleep apnea who were admitted to the intensive care unit (ICU) for 28 days. In addition, the univariate and multivariate logistic regression analyses of LAR and 28‐day mortality will be conducted.

### 3.2. Univariate and Multivariate Logistic Regression

The results of univariate and multivariate logistic regression analyses are presented in Table 4. Variables reaching statistical significance (*p* < 0.05) in univariate analysis, along with age and gender (retained for clinical relevance), were entered into the multivariate model. Multivariate analysis showed that the high‐LAR group had a higher risk of 28‐day mortality compared with the low‐LAR group (OR: 3.00; 95% CI: 1.83–4.90; *p* < 0.001). When LAR was analyzed as a continuous variable (per 1‐unit increase), the adjusted OR was 1.52 (95% CI: 1.27–1.82; *p* < 0.001), confirming a dose–response relationship between increasing LAR and higher 28‐day mortality.

**TABLE 4:** Univariate and multivariate logistic regression for 28‐day mortality.

Variable	Univariate OR (95% CI)	Univariate *p*	Multivariate OR (95% CI)	Multivariate *p*
Age (per year)	1.03 (1.02–1.05)	< 0.001	1.01 (0.99–1.03)	0.273
LAR (≥ 0.791 vs. < 0.791)	4.15 (2.89–5.95)	< 0.001	3.00 (1.83–4.90)	< 0.001
LAR (continuous, per unit)	—	—	1.52 (1.27–1.82)	< 0.001
OASIS (per point)	1.11 (1.08–1.13)	< 0.001	1.06 (1.02–1.09)	0.001
SAPSII (per point)	1.08 (1.06–1.09)	< 0.001	1.03 (1.01–1.06)	0.005
SOFA (per point)	1.20 (1.12–1.29)	< 0.001	0.97 (0.88–1.07)	0.558
Severe liver disease	2.45 (1.40–4.26)	0.002	2.62 (1.09–6.31)	0.031
Renal disease	1.62 (1.14–2.29)	0.007	1.06 (0.63–1.78)	0.823
MBP (per mmHg)	0.95 (0.93–0.97)	< 0.001	0.97 (0.94–1.00)	0.027
SpO2 (per %)	0.87 (0.78–0.96)	0.005	0.82 (0.71–0.95)	0.010
Base excess (per unit)	0.93 (0.90–0.96)	< 0.001	0.94 (0.86–1.02)	0.123

*Note:* MBP, mean arterial blood pressure.

Abbreviations: CI, confidence interval; LAR, lactate‐to‐albumin ratio; OASIS, Oxford Acute Severity of Illness Score; OR, odds ratio; SAPSII, Simplified Acute Physiology Score II; SOFA, Sequential Organ Failure Assessment.

#### 3.2.1. Subgroup Analysis

Figure 4 shows the results of the stratified analysis for the different subgroups, which include myocardial infarction, congestive heart failure, liver disease, diabetes, renal disease, malignant cancer, cerebrovascular disease, and chronic pulmonary disease. In all subgroups, a high level of LAR was significantly associated with an increased risk of mortality within 28 days (*p* < 0.05). There were no interactions in any of the subgroups (*p* value > 0.05).

**FIGURE 4:**
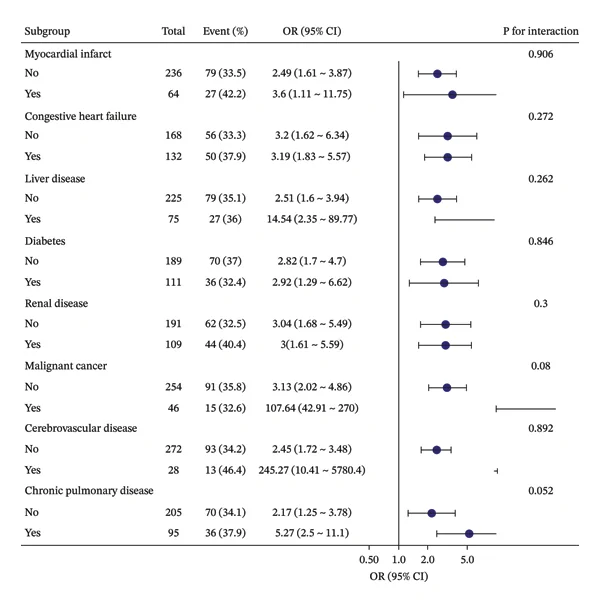
A subgroup analysis was conducted in order to establish the relationship between LAR and the risk of 28‐day mortality.

## 4. Discussion

This study constitutes the first large‐scale investigation of the association between LAR and 28‐day mortality in ICU patients with OSA, based on the MIMIC‐IV database. The present study demonstrated that LAR was an independent predictor of 28‐day mortality. The 28‐day mortality rate of patients in the high‐LAR group was found to be significantly higher than that of patients in the low‐LAR group. Despite adjustments for demographics, vital signs, laboratory indicators, and comorbidities, LAR demonstrated a persistent association with mortality (OR = 3.00, 95% CI: 1.83–4.90, *p* < 0.001). Importantly, this association remained significant when LAR was modeled as a continuous variable (adjusted OR = 1.52 per unit increase, 95% CI: 1.27–1.82, *p* < 0.001), supporting a graded relationship rather than a threshold effect alone. Furthermore, ROC and Kaplan–Meier curve analyses revealed that the predictive value of LAR for 28‐day mortality in OSA patients was essentially equivalent to that of OASIS and superior to SOFA. Moreover, subgroup analysis suggested that the association between LAR and 28‐day mortality remained consistent across all examined subgroups.

Previous retrospective studies involving medical, surgical, and mixed ICU settings have reported an OSA prevalence of 7.8% [17]. It is estimated that 38% of the global adult population suffers from moderate to severe OSA [1]. Moreover, OSA is not uncommon in critically ill patients, especially those with acute hypercapnic respiratory failure [18, 19]. It is well established that higher lactate concentrations in critically ill patients correspond to a higher risk of short‐term death [20]. Serum lactate is an indicator of tissue hypoxia. OSA causes hypoxemia and tissue hypoxia due to apneas during sleep; the resulting state of low oxygen delivery leads to hyperlactatemia. Increased respiratory rate causes respiratory muscle fatigue, further exacerbating lactate elevation [21]. Furthermore, under sleep‐related hypoxemic stress in OSA patients, pyruvate is converted to lactate via anaerobic glycolysis. Notably, plasma lactate levels increase in OSA patients but not in controls, and the magnitude of postsleep lactate elevation correlates with OSA severity and the degree of nocturnal hypoxemia. Serum lactate has thus been proposed as a marker of oxidative stress in OSA [22].

The prevalence of common comorbidities among ICU patients with OSA is high, including obesity, diabetes, hypertension, ischemic heart disease, heart failure, and cerebrovascular disease [17]. Hypoxemia is regarded as a cardiovascular risk factor that increases cardiac load and induces oxidative stress through the release of reactive oxygen species, thereby damaging vascular endothelial function [23], causing electrophysiological instability in the cardiac conduction system [24], and leading to severe metabolic disorders. Furthermore, OSA is very common among patients with chronic kidney disease (CKD) [25], and both experimental animal models [26, 27] and human studies [28–30] have reported that OSA contributes to CKD pathogenesis through intermittent hypoxia exposure. One study found that OSA significantly increased the incidence of acute kidney injury (AKI) by 124% [31]; compared with non‐OSA patients, the incidence of AKI was higher in OSA patients (41% vs. 57%, *p* < 0.001) [19]. Furthermore, previous studies have reported that nocturnal hypoxemia in OSA patients is associated with accelerated renal dysfunction [32]. OSA may cause accelerated renal function decline through multiple direct and indirect mechanisms, including hypoxia, sympathetic nervous system activation, hypertension, inflammation, oxidative stress, and renin–angiotensin–aldosterone system (RAAS) activation [33–36]. In an oxygen‐deficient environment, renal tubular epithelial cells mount inflammatory responses, triggering a cascade of pathological reactions including maladaptive cell repair, metabolic reprogramming, cell death/senescence, and fibrosis [37, 38]. Impaired kidney function can disrupt the body’s water and salt balance; airway congestion caused by excessive fluid accumulation may in turn play a role in the pathogenesis of sleep apnea [39].

Research has identified correlations between OSA and a range of cardiovascular diseases, metabolic disorders, and neurodegenerative diseases [40]. In this context, oxidative stress and inflammation caused by intermittent hypoxia appear to play a crucial role in OSA pathophysiology [41]. Serum albumin is an important antioxidant; structural modifications caused by free radicals can impair its antioxidant properties. It has been documented that in OSA, a decline in antioxidant capacity exacerbates oxidative stress, potentially precipitating cardiovascular and metabolic abnormalities [42]. Moreover, ischemia‐modified albumin (IMA)—a by‐product of albumin modification by free radicals—has been found to be markedly elevated in patients with severe OSA, and CPAP treatment appears to reverse this increase [43].

Multiple blood cell components and composite cell‐count indices have been identified as inflammatory markers in various inflammatory diseases, including OSA [41, 44–46]. However, only a limited number of studies have evaluated the prognostic ability of these indicators (mainly the neutrophil‐to‐lymphocyte ratio [NLR] and the platelet‐to‐lymphocyte ratio [PLR]), and results have been inconsistent [47, 48]. Therefore, through our investigation of the relationship between the lactate–albumin composite indicator and outcomes in ICU‐admitted OSA patients, we have demonstrated a significant correlation between LAR and OSA prognosis. This study presents a novel perspective on the risks associated with metabolic disruption in OSA, offering clinically relevant insights. Furthermore, LAR is a readily quantifiable indicator in clinical practice, with established reference ranges. As a composite indicator, LAR has been shown to reflect patient metabolic status more comprehensively than lactate or albumin alone. Renal insufficiency in OSA patients also reduces acidic metabolite excretion, further exacerbating acidosis. Consequently, LAR can be regarded as a significant prognostic marker for OSA patients, as it captures these pathophysiological changes.

### 4.1. Strengths and Limitations

Several strengths of this investigation should be acknowledged. First, LAR is a straightforward, noninvasive biomarker that requires minimal training to implement, making it readily applicable in primary healthcare settings. Second, our findings indicate that LAR exhibits moderate predictive value for 28‐day mortality in OSA patients, and the identified threshold may aid in risk stratification and timely intervention.

Several limitations must also be considered. First, as a retrospective post hoc analysis, selection bias and residual confounding are inherent. Notably, a large proportion of OSA patients (29,564 of 30,371) were excluded due to missing lactate or albumin values, which may introduce selection bias because these tests are typically ordered for clinically sicker patients. We acknowledge that our final cohort of 807 may not fully represent the broader ICU OSA population. Our study cohort mainly consists of patients who had lactate and albumin laboratory tests ordered by clinicians. Therefore, our conclusions cannot be extrapolated to all OSA patients admitted to the ICU. Second, certain parameters within the MIMIC‐IV database may be incomplete, potentially affecting data reliability. Although we addressed this by excluding variables with high missingness, a definitive solution remains elusive. Third, the study did not explore the influence of treatment strategies on LAR levels or their downstream effects on outcomes. Fourth, OSA was identified solely via ICD codes, which may not accurately reflect OSA severity or capture all cases, as polysomnography confirmation was not available for all patients. Finally, the present study did not account for the impact of therapeutic interventions on LAR and prognosis. Additionally, this study relies on a single‐center electronic database. Further external‐cohort validations, prospective multicenter investigations, and evaluations among non‐ICU patients with OSA are required to confirm the generalizability of our findings. Future research should further explore the potential value of LAR in guiding individualized treatment.

## 5. Conclusion

The results of this study indicate that LAR is significantly associated with 28‐day mortality in ICU patients with OSA. The underlying mechanism aligns with the physiological and metabolic processes of OSA. LAR demonstrates efficacy comparable to OASIS and superior to SOFA in predicting 28‐day mortality in patients diagnosed with OSA. Both categorical and continuous LAR analyses consistently support its independent prognostic value. As an easily obtainable and monitorable biomarker, LAR represents an important predictor of 28‐day mortality in this population.

## Author Contributions

Chuntian Chi had full access to all of the data in this study and takes complete responsibility for the integrity of the data and the accuracy of the data analysis. Jian Zhou: conceptualization, data curation, and original draft writing. Chuntian Chi: study design, statistical analysis, manuscript revision, and corresponding author. Junpei Wu, Fanghao Yu, and Hanjian Lou: data extraction, literature review, and manuscript editing. Jian Zhou and Chuntian Chi are the co‐first authors, who contributed equally to all experimental design, data collection, and manuscript writing.

## Funding

This study was financially supported by the Yiwu Science and Technology Plan Project (24‐3‐104).

## Disclosure

The corresponding author confirms that this manuscript is an honest, accurate, and transparent account of the study being reported; that no important aspects of the study have been omitted; and that any discrepancies from the study as planned have been explained. This is a retrospective observational cohort study and was not prospectively registered, consistent with the nature of database‐derived clinical research. The study was reported in accordance with the STROBE guidelines. The funding sources had no role in study design, data collection, analysis and interpretation, writing of the report, or the decision to submit the article for publication. All authors have read and approved the final version of the manuscript.

## Ethics Statement

The use of the MIMIC database was approved by the Institutional Review Board of the Massachusetts Institute of Technology and Beth Israel Deaconess Medical Center, both of which waive the need for informed consent for studies related to the MIMIC‐IV database; thus, our study did not require additional approval from the ethics committee of our own center. The study was conducted in accordance with the Declaration of Helsinki.

## Conflicts of Interest

The authors declare no conflicts of interest.

## Supporting Information

Additional supporting information can be found online in the Supporting Information section.

## Supporting information


**Supporting Information** The authors have completed the STROBE reporting checklist.

## Data Availability

The data that support the findings of this study are available from the corresponding author upon reasonable request.

## References

[1] Benjafield A. V. , Ayas N. T. , Eastwood P. R. et al., Estimation of the Global Prevalence and Burden of Obstructive Sleep Apnoea: A Literature-Based Analysis, Lancet Respiratory Medicine. (2019) 7, no. 8, 687–698, 10.1016/S2213-2600(19)30198-5. PMC7007763 31300334

[2] Luciano F. D. , Heno F. et al., The Impact of Obstructive Sleep Apnea on Metabolic and Inflammatory Markers in Consecutive Patients With Metabolic. Syndrome, PLoS One, 10.1371/journal.pone.0012065. PMC2920316 20711453

[3] Sun D. S. , Xu S. K. , Wang L. , Zhang L. , Yu H. Y. , and Shen J. Q. , The Weighted Combination of the Epworth Sleepiness Scale and the STOP-Bang Questionnaire Improved the Predictive Value of for OSAHS in Hypertensive Patients, International Journal of General Medicine. (2022) 15, 6909–6915, 10.2147/IJGM.S360544. PMC9439589 36061961

[4] Stepnowsky C. , Sarmiento K. F. , Bujanover S. , Villa K. F. , Li V. W. , and Flores N. M. , Comorbidities, Health-Related Quality of Life, and Work Productivity Among People With Obstructive Sleep Apnea With Excessive Sleepiness: Findings From the 2016 US National Health and Wellness Survey, Journal of Clinical Sleep Medicine. (2019) 15, no. 2, 235–243, 10.5664/jcsm.7624. PMC6374092 30736870

[5] Mulgrew A. T. , Ryan C. F. , Fleetham J. A. et al., The Impact of Obstructive Sleep Apnea and Daytime Sleepiness on Work Limitation, Sleep Medicine. (2007) 9, no. 1, 42–53, 10.1016/j.sleep.2007.01.009. 17825611

[6] Lal C. , Weaver T. E. , Bae C. J. , and Strohl K. P. , Excessive Daytime Sleepiness in Obstructive Sleep Apnea. Mechanisms and Clinical Management, Annals of the American Thoracic Society. (2021) 18, no. 5, 757–768, 10.1513/AnnalsATS.202006-696FR. PMC8086534 33621163

[7] Bender D. A. and Mayes P. A. , Murray R. K. and Granner D. K. , Glycolysis and the Oxidation of Pyruvate, Rodwell Vwharper’s Illustrated Biochemistry, 2006, 145, McGraw-Hill Education, Singapore.

[8] Kreisberg R. A. , Lactate Homeostasis and Lactic Acidosis, Annals of Internal Medicine. (1980) 92, no. 2_Part_1, 227–237, 10.7326/0003-4819-92-2-227. 6766289

[9] Bellomo R. , Bench-to-Bedside Review: Lactate and the Kidney, Critical Care. (2002) 6, 322–326, 10.1186/cc1518. PMC137458 12225607

[10] Alkozai E. M. , Mahmoodi B. K. , Decruyenaere J. et al., Systematic Comparison of Routine Laboratory Measurements With In-Hospital Mortality: Icu-Labome, a Large Cohort Study of Critically Ill Patients, Clinical Chemistry and Laboratory Medicine. (2018) 56, no. 7, 1140–1151, 10.1515/cclm-2016-1028. 29306908

[11] Masyuk M. , Wernly B. , Lichtenauer M. et al., Prognostic Relevance of Serum Lactate Kinetics in Critically Ill Patients, Intensive Care Medicine. (2019) 45, no. 1, 55–61, 10.1007/s00134-018-5475-3. 30478622

[12] Oua Y. , Hua J. , Wang Y. et al., Risk Analysis of the Association Between Red Blood Cell Distribution Width-to-Peripheral Oxygen Saturation Ratio and Poor Prognosis in Critical Patients. With Obstructive Sleep Apnea, Respiratory Medicine. (2025) 6, 10.1016/j.rmed.2025.108120. 40273998

[13] Jin X. , Li J. , Sun L. et al., Prognostic Value of Serum Albumin Level in Critically Ill Patients: Observational Data From Large Intensive Care Unit Databases, Frontiers in Nutrition. (2022) 9, 10.3389/fnut.2022.770674. PMC9234460 35769376

[14] Gharipour A. , Razavi R. , Gharipour M. , and Mukasa D. , Lactate/Albumin Ratio: An Early Prognostic Marker in Critically Ill Patients, The American Journal of Emergency Medicine. (2020) 38, no. 10, 2088–2095, 10.1016/j.ajem.2020.06.067. 33152585

[15] Zhang G. G. , Hao J. H. , Yong Q. et al., Lactate-to-Albumin Ratio is Associated With In-Hospital Mortality in Patients With Spontaneous Subarachnoid Hemorrhage and a Nomogram Model Construction, Frontiers in Neurology. (2022) 13, 10.3389/fneur.2022.1009253. PMC9618723 36324387

[16] Xu C. , Liu H. , Zhang H. et al., Predictive Value of Arterial Blood Lactate to Serum Albumin Ratio for In-Hospital Mortality of Patients With Community-Acquired Pneumonia Admitted to the Intensive Care Unit, Postgraduate Medical Journal. (2022) 135, no. 3, 1–10, 10.1080/00325481.2022.2110769. 35930266

[17] Bolona E. , Hahn P. Y. , and Afessa B. , Intensive Care Unit and Hospital Mortality in Patients With Obstructive Sleep Apnea, Journal of Critical Care. (2015) 30, no. 1, 178–180, 10.1016/j.jcrc.2014.10.001. 25457113

[18] Thille A. W. , Córdoba-Izquierdo A. , Maitre B. , Boyer L. , Brochard L. , and Drouot X. , High Prevalence of Sleep Apnea Syndrome in Patients Admitted to ICU for Acute Hypercapnic Respiratory Failure: A Preliminary Study, Intensive Care Medicine. (2018) 44, no. 2, 267–269, 10.1007/s00134-017-4998-3. 29159561

[19] Dou L. , Lan H. , Reynolds D. J. et al., Association Between Obstructive Sleep Apnea and Acute Kidney Injury in Critically Ill Patients: A Propensity-Matched Study, Nephron. (2017) 135, no. 2, 137–146, 10.1159/000453367. 27951537

[20] Vincent J. L. , Quintairos E. S. A. , Couto L. , and Taccone F. S. , The Value of Blood Lactate Kinetics in Critically Ill Patients: A Systematic Review, Critical Care. (2016) 20, no. 1, 10.1186/s13054-016-1403-5. PMC4983759 27520452

[21] Ranucci M. , De Toffol B. , Isgro G. , Romitti F. , Conti D. , and Vicentini M. , Hyperlactatemia During Cardiopulmonary Bypass: Determinants and Impact on Postoperative Outcome, Critical Care. (2006) 10, no. 6, 10.1186/cc5113. PMC1794481 17134504

[22] Singh Hira H. , Shukla A. , and Kaur A. , Serum Uric Acid and Lactate Levels Among Patients With Obstructive Sleep Apnea Syndrome, Which?. (2012) 12012, no. 37, 10–5144, 10.5144/0256-4947. PMC6087645 22156638

[23] Takabatake N. , Nakamura H. , Abe S. et al., The Relationship Between Chronic Hypoxemia and Activation of the Tumor Necrosis Factor-Alpha System in Patients With Chronic Obstructive Pulmonary Disease, American Journal of Respiratory and Critical Care Medicine. (2000) 161, no. 4, 1179–1184, 10.1164/ajrccm.161.4.9903022. 10764309

[24] Zamarron C. , Garcia Paz V. , Morete E. et al., Association of Chronic Obstructive Pulmonary Disease and Obstructive Sleep Apnea Consequences, International Journal of Chronic Obstructive Pulmonary Disease. (2008) 671–682, 10.2147/COPD.S4950. PMC2650593 19281082

[25] Nicholl D. D. M. , Ahmed S. B. , Loewen A. H. S. et al., Declining Kidney Function Increases the Prevalence of Sleep Apnea and Nocturnal Hypoxia, Chest. (2012) 141, no. 6, 1422–1430, 10.1378/chest.11-1809. 22222188

[26] Evans R. G. , Smith D. W. , Lee C. J. et al., Factors that Render the Kidney Susceptible to Tissue Hypoxia in Hypoxemia, American Journal of Physiology–Regulatory, Integrative and Comparative Physiology. (2011) 300, 931–940, 10.1152/ajpregu.00552.2010. 21248306

[27] Abuyassin B. , Badran M. , Ayas N. T. , and Laher I. , Intermittent Hypoxia Causes Histological Kidney Damage and Increases Growth Factor Expression in a Mouse Model of Obstructive Sleep Apnea, PLoS One. (2018) 13, no. 2, 10.1371/journal.pone.0192084. PMC5794148 29389945

[28] Foster G. E. , Hanly P. J. , Ahmed S. B. , Beaudin A. E. , Pialoux V. , and Poulin M. J. , Intermittent Hypoxia Increases Arterial Blood Pressure in Humans Through a Renin-Angiotensin System-Dependent Mechanism, Hypertension. (2010) 56, no. 3, 369–377, 10.1161/HYPERTENSIONAHA.110.15210. 20625082

[29] Zalucky A. A. , Nicholl D. D. M. , Hanly P. J. et al., Nocturnal Hypoxemia Severity and Renin-Angiotensin System Activity in Obstructive Sleep Apnea, American Journal of Respiratory and Critical Care Medicine. (2015) 192, no. 7, 873–880, 10.1164/rccm.201502-0383OC. 26102156

[30] Nicholl D. D. M. , Hanly P. J. , Zalucky A. A. , Handley G. B. , Sola D. Y. , and Ahmed S. B. , Nocturnal Hypoxemia Severity Influences the Effect of CPAP Therapy on Renal Renin-Angiotensin-Aldosterone System Activity in Humans With Obstructive Sleep Apnea, Sleep. (2021) 44, no. 5, 10.1093/sleep/zsaa228. 33734385

[31] Wang J. , Wang X. , Yu W. , Zhang K. , and Wei Y. , Obstructive Sleep Apnea-Induced Multi-Organ Dysfunction After Elective Coronary Artery Bypass Surgery in Coronary Heart Disease Patients, Journal of Thoracic Disease. (2020) 12, no. 10, 5603–5616, 10.21037/jtd-20-2037. PMC7656408 33209393

[32] Ahmed S. B. , Ronksley P. E. , Hemmelgarn B. R. et al., Nocturnal Hypoxia and Loss of Kidney Function, PLoS One. (2011) 6, no. 4, 10.1371/journal.pone.0019029. PMC3084745 21559506

[33] Sun W. , Yin X. , Wang Y. et al., Intermittent Hypoxia-Induced Renal Antioxidants and Oxidative Damage in Male Mice: Hormetic Dose Response, Dose-Response. (2012) 11, no. 3, 385–400, 10.2203/dose-response.12-027. PMC3748850 23983666

[34] Brooks D. , Horner R. L. , Kozar L. F. , Render-Teixeira C. L. , and Phillipson E. A. , Obstructive Sleep Apnea as a Cause of Systemic Hypertension. Evidence From a Canine Model, Journal of Clinical Investigation. (1997) 99, no. 1, 106–109, 10.1172/jci119120. PMC507773 9011563

[35] Somers V. K. , Dyken M. E. , Clary M. P. , and Abboud F. M. , Sympathetic Neural Mechanisms in Obstructive Sleep Apnea, Journal of Clinical Investigation. (1995) 96, no. 4, 1897–1904, 10.1172/jci118235. PMC185826 7560081

[36] Ryan S. , Taylor C. T. , and McNicholas W. T. , Selective Activation of Inflammatory Pathways by Intermittent Hypoxia in Obstructive Sleep Apnea Syndrome, Circulation. (2005) 112, no. 17, 2660–2667, 10.1161/circulationaha.105.556746. 16246965

[37] Wang B. , Li Z. L. , Zhang Y. L. , Wen Y. , Gao Y. M. , and Liu B. C. , Hypoxia and Chronic Kidney Disease, EBioMedicine. (2022) 77, 10.1016/j.ebiom.2022.103942. PMC8921539 35290825

[38] Shu S. , Wang Y. , Zheng M. et al., Hypoxia and Hypoxia-Inducible Factors in Kidney Injury and Repair, Cells. (2019) 8, no. 3, 10.3390/cells8030207. PMC6468851 30823476

[39] Tang S. C. , Lam B. , Lai A. S. et al., Improvement in Sleep Apnea During Nocturnal Peritoneal Dialysis is Associated With Reduced Airway Congestion and Better Uremic Clearance, Clinical Journal of the American Society of Nephrology. (2009) 4, no. 2, 410–418, 10.2215/cjn.03520708. PMC2637593 19118118

[40] Meliante P. G. , Zoccali F. , Cascone F. et al., Molecular Pathology, Oxidative Stress, and Biomarkers in Obstructive Sleep Apnea, International Journal of Molecular Sciences. (2023) 24, no. 6, 10.3390/ijms24065478. PMC10058074 36982552

[41] Suzuki Y. J. , Jain V. , Park A. M. , and Day R. M. , Oxidative Stress and Oxidant Signaling in Obstructive Sleep Apnea and Associated Cardiovascular Diseases, Free Radical Biology and Medicine. (2006) 40, no. 10, 1683–1692, 10.1016/j.freeradbiomed.2006.01.008. PMC1995030 16678006

[42] Faure P. , Tamisier R. , Baguet J. P. et al., Impairment of Serum Albumin Antioxidant Properties in Obstructive Sleep Apnoea Syndrome, European Respiratory Journal. (2008) 31, no. 5, 1046–1053, 10.1183/09031936.00062707. 18256067

[43] Düger M. , Seyhan E. C. , Günlüoğlu M. Z. et al., Does Ischemia-Modified Al-Bumin Level Predict Severity of Obstructive Sleep Apnea?, Sleep and Breathing. (2021) 25, no. 1, 65–73, 10.1007/s11325-020-02038-9. 32212064

[44] Paliogiannis P. , Fois A. G. , Sotgia S. et al., The Neutrophil-to-Lymphocyte Ratio as a Marker of Chronic Obstructive Pulmonary Disease and Its Exacerbations: A Systematic Review and Meta-Analysis, European Journal of Clinical Investigation. (2018) 48, no. 8, 10.1111/eci.12984. 29924383

[45] Kıvanc T. , Kulaksızoglu S. , Lakadamyal H. et al., Importance of Laboratory Parameters in Patients With Obstructive Sleep Apnea and Their Relationship With Cardiovascular Diseases, Journal of Clinical Laboratory Analysis. (2018) 32, 10.1002/jcla.22199. PMC6816860 28345811

[46] Putzu C. , Cortinovis D. L. , Colonese F. et al., Blood Cell Count Indexes as Predictors of Outcomes in Advanced Non-Small-Cell Lung Cancer Patients Treated With Nivolumab, Cancer Immunology Immunotherapy. (2018) 67, no. 9, 1349–1353, 10.1007/s00262-018-2182-4. PMC11028046 29947960

[47] Pinna A. , Porcu T. , Paliogiannis P. et al., Complete Blood Cell Count Measures in Retinal Artey Occlusions, Acta Ophthalmologica. (2021) 99, no. 6, 637–643, 10.1111/aos.14699. 33629472

[48] Rha M. S. , Kim C. H. , Yoon J. H. , and Cho H. J. , Association Between the Neutrophil-to-Lymphocyte Ratio and Obstructive Sleep Apnea: A Meta-Analysis, Scientific Reports. (2020) 10, no. 1, 10.1038/s41598-020-67708-w. PMC7331605 32616762

