# Supplementary material for: Association of Lactate‐to‐Albumin Ratio With 28‐Day Mortality in Critically Ill Patients With Obstructive Sleep Apnea: A Retrospective Cohort Study Based on the MIMIC‐IV Database
Source: Can Respir J. 2026 Nov 5;2026:6582764. doi: 10.1155/carj/6582764 (PMC13735216; doi:10.1155/carj/6582764)
Supplement: Supplementary file 1 [file CARJ-2026-6582764-s001.pdf]

STROBE Statement—checklist of items that should be included in reports of observational studies

Manuscript: Association of Lactate-to-Albumin Ratio with 28-day Mortality in Critically Ill Patients with Obstructive Sleep Apnea: a Retrospective Cohort Study Based on the MIMIC-IV Database

Study design: Retrospective cohort study (STROBE-compliant reporting)

Item No	Recommendation	Reported on page No
**Title and abstract**		
1 (a)	Indicate the study's design with a commonly used term in the title or the abstract	Title
1 (b)	Provide in the abstract an informative and balanced summary of what was done and what was found	Abstract
**Introduction**		
2	Explain the scientific background and rationale for the investigation being reported	Introduction (paragraphs 1–3)
3	State specific objectives, including any prespecified hypotheses	Introduction (final paragraph); Abstract
**Methods**		
4	Present key elements of study design early in the paper	Abstract (Methods); Methods (Database)
5	Describe the setting, locations, and relevant dates, including periods of recruitment, exposure, follow-up, and data collection	Methods (Database)
6 (a)	Cohort study—Give the eligibility criteria, and the sources and methods of selection of participants. Describe methods of follow-up<br>Case-control study—Give the eligibility criteria, and the sources and methods of case ascertainment and control selection. Give the rationale for the choice of cases and controls<br>Cross-sectional study—Give the eligibility criteria, and the sources and methods of selection of participants	Methods (Study Population); Figure 1
6 (b)	Cohort study—For matched studies, give matching criteria and number of exposed and unexposed<br>Case-control study—For matched studies, give matching criteria and the number of controls per case	N/A (unmatched cohort study)
7	Clearly define all outcomes, exposures, predictors, potential confounders, and effect modifiers. Give diagnostic criteria, if applicable	Methods (Data Extraction; Statistical Analysis)
8*	For each variable of interest, give sources of data and details of methods of assessment (measurement). Describe comparability of assessment methods if there is more than one group	Methods (Data Extraction)
9	Describe any efforts to address potential sources of bias	Methods (Statistical Analysis); Discussion (Limitations)
10	Explain how the study size was arrived at	Methods (Study Population)

Item No	Recommendation	Reported on page No
11	Explain how quantitative variables were handled in the analyses. If applicable, describe which groupings were chosen and why	Methods (Statistical Analysis)
12 (a)	Describe all statistical methods, including those used to control for confounding	Methods (Statistical Analysis)
12 (b)	Describe any methods used to examine subgroups and interactions	Methods (Statistical Analysis)
12 (c)	Explain how missing data were addressed	Methods (Study Population); Table 2
12 (d)	Cohort study—If applicable, explain how loss to follow-up was addressed<br>Case-control study—If applicable, explain how matching of cases and controls was addressed<br>Cross-sectional study—If applicable, describe analytical methods taking account of sampling strategy	N/A (complete 28-day follow-up; no loss to follow-up)
12 (e)	Describe any sensitivity analyses	Not reported (no sensitivity analyses performed)
**Results**		
13 (a)*	Report numbers of individuals at each stage of study—eg numbers potentially eligible, examined for eligibility, confirmed eligible, included in the study, completing follow-up, and analysed	Methods (Study Population); Figure 1
13 (b)*	Give reasons for non-participation at each stage	Methods (Study Population)
13 (c)*	Consider use of a flow diagram	Figure 1
14 (a)*	Give characteristics of study participants (eg demographic, clinical, social) and information on exposures and potential confounders	Results; Table 1
14 (b)*	Indicate number of participants with missing data for each variable of interest	Methods (Study Population); Table 2
14 (c)*	Cohort study—Summarise follow-up time (eg, average and total amount)	Results (28-day follow-up; 28-day mortality 20.44%)
15*	Cohort study—Report numbers of outcome events or summary measures over time<br>Case-control study—Report numbers in each exposure category, or summary measures of exposure<br>Cross-sectional study—Report numbers of outcome events or summary measures	Results; Figure 3
16 (a)	Give unadjusted estimates and, if applicable, confounder-adjusted estimates and their precision (eg, 95% confidence interval). Make clear which confounders were adjusted for and why they were included	Table 4; Methods (Statistical Analysis)
16 (b)	Report category boundaries when continuous variables were categorized	Table 3; Results (ROC analysis)
16 (c)	If relevant, consider translating estimates of relative risk into absolute risk for a meaningful time period	N/A (odds ratios reported)

Item No	Recommendation	Reported on page No
17	Report other analyses done—eg analyses of subgroups and interactions, and sensitivity analyses	Figure 4
**Discussion**		
18	Summarise key results with reference to study objectives	Discussion (first paragraph)
19	Discuss limitations of the study, taking into account sources of potential bias or imprecision. Discuss both direction and magnitude of any potential bias	Discussion (Strengths and Limitations)
20	Give a cautious overall interpretation of results considering objectives, limitations, multiplicity of analyses, results from similar studies, and other relevant evidence	Discussion (paragraphs 2–6)
21	Discuss the generalisability (external validity) of the study results	Discussion (Limitations); Conclusion
**Other information**		
22	Give the source of funding and the role of the funders for the present study and, if applicable, for the original study on which the present article is based	Funding statement

*Give information separately for cases and controls in case-control studies and, if applicable, for exposed and unexposed groups in cohort and cross-sectional studies.

Note: An Explanation and Elaboration article discusses each checklist item and gives methodological background and published examples of transparent reporting. The STROBE checklist is best used in conjunction with this article (freely available on the Web sites of PLoS Medicine at http://www.plosmedicine.org/, Annals of Internal Medicine at http://www.annals.org/, and Epidemiology at http://www.epidem.com/). Information on the STROBE Initiative is available at www.strobe-statement.org.

Location references in the "Reported on page No" column indicate the manuscript sections, tables and figures where each item is addressed; update with the final page numbers after typesetting if required by the journal.
